# Regional microglial activation differs across the cortex of patients in the Alzheimer's disease trajectory: a cross‐sectional study

**DOI:** 10.1002/alz.094039

**Published:** 2025-01-09

**Authors:** Marie Emilie Tuil, Valeria Calsolaro, Harry Crook, Grazia Daniela Femminella, Giuseppe Pasqualetti, Christopher Buckley, William Trigg, Steve Gentleman, David J Brooks, Simona Jogaudaite, Javier Alegre Abarrategui, Rainer Hinz, Paul Edison

**Affiliations:** ^1^ Imperial College London, London United Kingdom; ^2^ GE HealthCare, Amersham United Kingdom; ^3^ GE Healthcare, Amersham United Kingdom; ^4^ University of Manchester, Manchester United Kingdom

## Abstract

**Background:**

Microglial reactivity and neuroinflammation are crucial pathological processes in Alzheimer’s Disease (AD). Several attempts to develop a treatment by supressing the immune response in AD have been made, yet these yielded very limited results. Recent studies suggest contrasting effects of microglial reactivity, indicating a biphasic response with both beneficial and deleterious effects at distinct stages of AD. We evaluated the regional differential influence of microglial reactivity on neuropathological processes across AD trajectory.

**Method:**

48 patients were divided by diagnosis into control (11), Mild Cognitive Impairment (MCI, 14), and AD (23) groups. They underwent TSPO‐PET imaging, magnetic resonance imaging (MRI), and neuropsychological testing. The spectral analysis generated a TSPO‐PET impulse response function at 90 minutes (IRF90), reflecting its volume of distribution. Region of interest and voxel‐level analyses were performed, and grey matter (GM) volume was calculated using FreeSurfer and voxel‐based morphometric analysis. Additionally, post mortem brain tissue from 27 patients at Braak Stages IV‐VI was stained for CD32a and CD163, markers of pro and anti‐inflammatory activation respectively.

**Result:**

The AD group showed increased microglial reactivity in the temporal, parietal, occipital, and medio‐temporal lobes, and the cingulate cortex compared to controls. Voxel‐wide analyses of AD and MCI patients yielded similar results across the cortex. The voxel‐wise analysis revealed both positive and negative clusters of association between tracer uptake and GM volume, with negative associations more often found in regions affected earlier in AD trajectory. This dual effect is further denoted by the presence of both CD32a‐positive and CD164‐positive microglia within the same regions in post mortem tissue.

**Conclusion:**

The contrasting effects of reactive microglia on different pathological processes show regional disparities, with deleterious effects more closely associated with regions affected in the early stages of AD. This suggests a diverging response driven by both anti‐ and pro‐inflammatory reactivity profiles. The former, neuroprotective, would emerge locally alongside early pathology, while the later, neurotoxic, would only appear with more advanced pathology. Consequently, potential treatments should promote anti‐inflammatory polarization and supress pro‐inflammatory activation in microglia for an effective therapeutic strategy.

